# SDF-1/CXCL12: A Chemokine in the Life Cycle of HIV

**DOI:** 10.3389/fimmu.2015.00256

**Published:** 2015-06-05

**Authors:** Fernando Arenzana-Seisdedos

**Affiliations:** ^1^INSERM U1108, Laboratory of Viral Pathogenesis, Institut Pasteur, Paris, France

**Keywords:** chemokine, SDF-1, CXCL12, HIV, coreceptor, viral entry

## In the Arena of HIV-1 Research

Since 1986, the beginning of our involvement in HIV research, our major interest was focused on the regulation of HIV-1 replication by the transcriptional host cell machinery. Thus, we successively investigated the consequences of inflammatory and specific responses in HIV replication in primary monocytes and memory CD4^+^ T cells, and explored with special emphasis the role played by the transcriptional factors NF-kB and the viral trans-activator Tat in the induction and maintaining of the activity of HIV-1 promoter region, while in parallel we elucidated some of the critical mechanisms leading to the activation of NF-kB factors.

Our initial interest in chemokines was based on our hypothesis that they could act both as chemoattractants for HIV-1 target cells and inducers of HIV-1 transcription and replication. During our early chemokine studies, the group of Paolo Lusso reported in 1995 that the CC chemokines CCL5/RANTES, CCL3/MIP-1α, and CCL4/Mip-1β, isolated from an immortalized CD8^+^ T lymphocyte clone, blocked infection of a CD4^+^ T cell line susceptible to primary HIV-1 isolates and some HIV-2 and SIV isolates (1). Based on the tight relationship between T cell activation and HIV replication, the blockade of HIV infection by these factors was in an apparent contradiction with the strong and recently reported potent antigen-independent activation in T lymphocytes by RANTES.

## The Converging Paths of HIV-1 Entry and Chemokine Research

The HIV inhibitory effect of the chemokines identified by Paolo Lusso’s group was associated to the previously known, although poorly characterized, suppressive effect of CD8^+^ T lymphocytes culture supernatants (1, 2). However, the hypothesis of a possible interference of this mechanism on HIV entry was not raised in the report. HIV entry in CD4 T lymphocytes was known to critically rely on the interaction of the HIV envelope glycoproteins (surface subunit gp120) with CD4, a viral receptor and a critical determinant of viral tropism, in that gp120 binding to CD4 eventually leads to viral/target cell membrane fusion and entry of viral replication machinery. Importantly, this early research clearly established that an essential cofactor for HIV entry was missing as CD4 alone did not support HIV infection. While many teams all over the world were trying unsuccessfully to identify such CD4 cofactor(s) enabling productive infection by HIV, scientist working in the field of chemokines were making tremendous progress identifying new chemokines and receptors and elucidating their biological roles.

Among them, Bernhard Moser at the Theodor-Kocher Institute in Bern, directed by Marco Baggiolini, had isolated the cDNA for the orphan receptor LESTR, which shared typical characteristics of G protein-coupled receptors (GPCRs) (2). Although the ligand for LESTR could not be identified among a large number of identified chemotactic cytokines, the high expression in white blood cells and the marked sequence relation to CXCR1/IL-8R1 and CXCR2/IL-8R2 suggested that LESTR may be a novel receptor for an unknown chemokine. In a collaboration with Conrad Bleul, a post-doctoral scientist in Tim Springer’s laboratory at Harvard University, Bernhard obtained preliminary evidence indicating that LESTR is the selective chemokine receptor for the so-called Pre-B-cell growth-stimulating factor/stromal cell-derived factor 1 (PBSF/SDF-1/CXCL12), a member of the C–X–C subfamily, which was originally cloned by two independent groups (3, 4). This highly conserved chemokine turned out to have an essential (non-redundant) role in B cell lymphopoiesis as well as the normal development of the heart, the vasculature, and the brain. The characterization of tissue expression of CXCL12/SDF-1 was first shown in mice and we provided the first tissue expression mapping in humans (5). In keeping with the high degree of homology, the mouse sample of active CXCL12/SDF-1 that Conrad Bleul brought to the Theodor-Kocher Institute behaved as an agonist on cells expressing human LESTR.

Another breakthrough in HIV research was announced in early 1996 at a Keystone Symposium by Edward Berger from the National Institute of Health in Bethesda who reported unpublished findings about a novel cell fusion coreceptor allowing the infection by T cell line-tropic (TCL-tropic or T-tropic) but not macrophage-tropic (M-tropic) HIV-1 isolates when co-expressed on CD4^+^ target cells. In the subsequent paper by Ed Berger’s group (6), this fusion coreceptor was called “fusin” and turned out to be identical to LESTR that Bernhard’s group was working on. The ground-breaking discovery by Ed Berger’s group revealed the definitive link between the interaction of HIV-1 and its target cells and the rapidly expanding field of chemokine receptors.

## CXCL12/CXCR4 and HIV-1 Infection: The Meeting Point

In early 1996, scientists from the Theodor-Kocher Institute have already been engaged in collaborations with groups at the Pasteur Institute in Paris who were investigating host immune cell responses to parasites and the “fusin” discovery led to joint efforts of our laboratories in the new arena of HIV research. This was the beginning of a very fruitful and effective collaboration between the scientists at the Theodor-Kocher Institute in Bern and our department at the Pasteur Institute in Paris.

**Figure 1:**
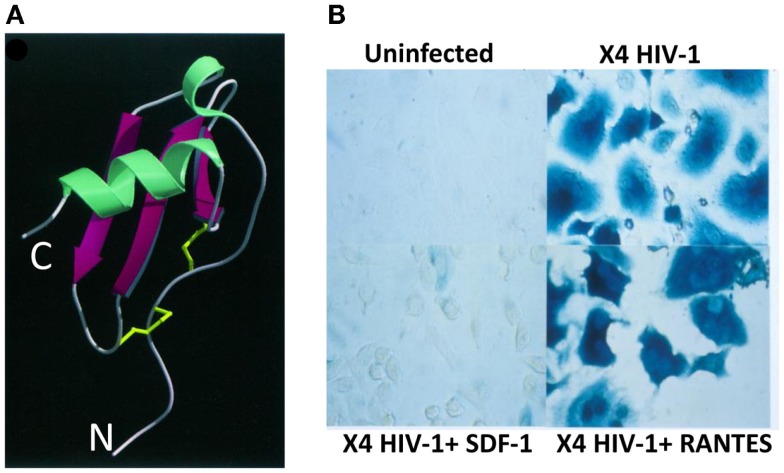
***Structure and mode of action of CXCL12/SDF-1 in HIV-1 infection***. **(A)** Ribbon structure of human CXCL12/SDF-1α. The two disulfur bonds are indicated in yellow (9). N, amino-terminus; C, carboxy-terminus. **(B)** Human cells expressing CD4 and CXCR4 infected or not with an X4 HIV-1 isolate in the presence of SDF-1/CXCL12 or RANTES/CCL5. Infected cells are visualized by β-galactosidase staining.

Thus, we immediately confirmed Bernhard’s findings about LESTR being the specific receptor for CXCL12/SDF-1 using both CHO cell lines transduced to express LESTR and human leukocytes, including neutrophils, monocytes, and T lymphocytes. In all these cell types, CXCL12/SDF-1-induced robust chemotaxis and intracellular Ca^2+^ responses, which are typical responses seen with chemokines binding of cognate receptors. Simultaneously, our findings were confirmed by Conrad Bleul at Harvard University and, consequently, LESTR was renamed as CXCR4, the fourth CXC chemokine receptors to be identified. Also, both groups obtained simultaneously evidence that CXCL12/SDF-1 prevented selectively the infection by T-tropic HIV isolates (laboratory adapted or primary) but was unable to prevent infection of activated T lymphocytes by M-tropic viral isolates (7, 8). However, as we demonstrated, CC chemokines CCL3/MIP-1α, CCL4/MIP-1β, and CCL5/RANTES were unable to block infection by T-tropic isolates thus proving the specific and selective inhibition by CXCL12/SDF-1 for this type of viruses (Figure 1). Moreover, Conrad Bleul’s work demonstrated that only CXCL12/SDF-1 including an intact amino-terminal domain was active as suppressor factor for infection by T-tropic viruses, indicating that the amino-terminal domain in CXCL12/SDF-1 was required for both activation of CXCR4 and inhibition of T-tropic HIV-1 species. This experiment suggested that CXCL12/SDF-1 could inhibit HIV infection by steric hindrance of viral pg120 binding to CXCR4. We also showed that CXCL12/SDF-1 blocked T-tropic HIV-1 infection at an early step without affecting the rest of the HIV-1 life cycle by setting up two complementary experiments (7). First, we demonstrated that CXCL12/SDF-1 potently prevented the accumulation newly reverse-transcribed HIV proviral DNA from the genomic viral RNA, a mandatory process required for productive infection. Second, using HIV-1 particles whose envelope glycoprotein was replaced by the one from vesicular stomatitis virus, which enables infection in a CD4- and coreceptor-independent manner, we proved that CXCL12/SDF-1 failed to inhibit the viral replication (occurring after viral entry). Both sets of findings described above were published as back-to-back papers in August 1996.

Obviously, CXCL12/SDF-1 was an excellent new target for the development of novel inhibitors of infection by T-tropic HIV-1 species. Based on a collaboration that included Ian Clark-Lewis from the University of British Columbia, we carried out detailed structure–function studies and came up with a two-site model for binding of CXCL12/SDF-1 to CXCR4, which became a general a model for chemokine binding to their cognate receptors (9). Similar work with CCL5/RANTES led to the identification of CCR5 derivatives that failed to induce chemokine responses in leukocytes yet retained the ability to block HIV-1 entry of M-tropic but not T-tropic viruses (10). Simultaneously, we reported that the anti-HIV-1 blocking activity of CXCL12/CXCL12 critically involved the cell surface depletion of CXCR4, a phenomenon that was mediated by the intracellular carboxy-terminal region of CXCR4 in a G protein-independent fashion (11).

## Following the Track: Chemokines and HIV-1 Some Years Later

The accumulation of native chemokines that bind CCR5 with high affinity (R5-CHKs) into the anatomical sites of HIV replication suggests that they could act as a natural barrier against HIV infection, both by displacing the viral envelope glycoprotein gp120 from binding to CCR5 and by promoting CCR5 endocytosis. However, the concentration of R5-CHKs required to inhibit R5 HIV-1 primary strains in primary CD4 cell targets exceed by several orders of magnitude the receptor $K_{\text{d}}^{\text{s}}$ value and chemotaxis-induction, a G-protein-dependent mechanism. A possible explanation for this intriguing phenomenon is now provided by recent work from our laboratory (12). We showed that different CCR5 conformations at the cell surface are differentially engaged by R5-CHKs and gp120, making R5-CHKs weaker inhibitors of HIV infection than would be expected from their binding affinity constants for CCR5. These distinct CCR5 conformations rely on CCR5 coupling to G-proteins. While R5-CHKs bind with high affinity (*K*_d_ < 1 nM) to active conformations of CCR5 coupled to nucleotide-free G-proteins (NFG), gp120/HIV-1 does not discriminate between NFG-protein coupled an uncoupled CCR5. Interestingly, the antiviral activity of R5-CHKs is G-protein independent, suggesting that inactive CCR5, which are of low affinity for R5-CHKs, represent a portal for viral entry. This is reminiscent of infection by R5 HIV-1, which occurs also in a G-protein-independent fashion (13). Furthermore, R5-CHKs are weak inducers of CCR5 endocytosis, as is revealed by their potencies in the submicromolar range for inducing endocytosis reflecting their low-affinity constant value for NFG-protein-uncoupled receptors. Abolishing CCR5 interaction with NFG-proteins eliminates high-affinity binding of R5-CHKs but preserves receptor endocytosis, indicating that R5-CHKs preferentially endocytose low-affinity receptors. These data are consistent with HIV-1 evading R5-CHK inhibition by exploiting CCR5 conformations that are weakly recognized by native chemokines, named “spare receptors” that are unlikely to take part in R5-CHKs-mediated functional responses. Importantly, and in contrast to native chemokines, some RANTES/CCR5 antagonists and agonist analogs displaying improved anti-HIV-1 activity recognize this fraction of CCR5 receptors, thus proving the importance of blocking “spare receptors” for preventing HIV-1 infection (14).

By sharp contrast, the affinity of CXCL12/SDF-1 for CXCR4 correlates well with its HIV-1 inhibitory activity and its ability to induce CXCR4 internalization. This property could explain the selective CXCR4 down-modulation on intestinal lymphocytes in response to local CXCL12 constitutively produced by gut epithelia (15). Mucosal epithelia are a site of prominent HIV-1 replication and local CXCL12/SDF-1 could in part explain the observed predominance of M-tropic HIV-1 variants, which are not affected by CXCL12/SDF-1.

## Conclusion

The seminal work reported by the laboratories of Paolo Lusso and Ed Berger initiated an unprecedented storm of collaborative activities across the fields of chemokine and HIV research. It is now firmly established that CCR5 and CXCR4 are the principal coreceptors for M-tropic and T-tropic HIV-1 variants (also referred to as R5 and X4 HIV variants), respectively. Maraviroc, a CCR5-specific antagonist, is currently used in the treatment of HIV infected individuals. Still, many questions remain. For instance, R5 HIV-1 viruses are transmitted and propagated preferentially during the early and asymptomatic stages of infection while viruses showing CXCR4 tropism (X4 HIV-1 and, mainly, dual tropic X4R5 HIV-1) emerge progressively and become detectable in roughly 40–50% of infected people at later stages of the infection or during the AIDS phase. This apparent paradox is still unresolved, as CXCR4 expression is constitutive and ubiquitous, including most nucleated cells and, most notably, CD4^+^ T cells. By clear contrast, expression of CCR5 is restricted to activated effector T cells, which are a minor subset of T cells in peripheral blood, and dendritic cells indicating that target cells for R5 HIV-1 are much more limited. The causes underlying this phenomenon are likely multifactorial and a number of possible mechanisms had been proposed. The fact that X4 HIV-1 viruses rapidly emerge in a significant proportion of HIV-1-infected patients treated by the CCR5-specific antagonist maraviroc and spontaneously regress as the administration of this drug is interrupted, suggests that a certain degree of competition between R5 and X4 HIV-1 viruses exists.

The hectic research activities carried out during the first half of 1996 was due to intense collaborations set up by research teams working in, *a priori*, separated fields such as molecular virology, chemokine biology, or GPCR pharmacology. Within this setting, the real contribution of chemokine and chemokine receptor research to the new field was that it progressively implemented and transformed our basic knowledge of HIV cell tropism into a detailed view and understanding of the complex molecular mechanisms of HIV entry leading to novel therapeutic strategies for blocking HIV infection.

## Conflict of Interest Statement

The author declares that the research was conducted in the absence of any commercial or financial relationships that could be construed as a potential conflict of interest.

## References

[1] Cocchi F DeVico AL Garzino-Demo A Arya SK Gallo RC Lusso P. Identification of RANTES, MIP-1 alpha, and MIP-1 beta as the major HIV-suppressive factors produced by CD8+ T cells. Science (1995) 270(5243):1811–5. 10.1126/science.270.5243.1811 8525373

[2] Loetscher M Geiser T O’Reilly T Zwahlen R Baggiolini M Moser B. Cloning of a human seven-transmembrane domain receptor, LESTR, that is highly expressed in leukocytes. J Biol Chem (1994) 269(1):232–7. 8276799

[3] Nagasawa T Kikutani H Kishimoto T. Molecular cloning and structure of a pre-B-cell growth-stimulating factor. Proc Natl Acad Sci U S A (1994) 91(6):2305–9. 10.1073/pnas.91.6.2305 8134392 PMC43359

[4] Tashiro K Tada H Heilker R Shirozu M Nakano T Honjo T. Signal sequence trap: a cloning strategy for secreted proteins and type I membrane proteins. Science (1993) 261(5121):600–3. 10.1126/science.8342023 8342023

[5] Coulomb-L’Hermin A Amara A Schiff C Durand-Gasselin I Foussat A Delaunay T Stromal cell-derived factor 1 (SDF-1) and antenatal human B cell lymphopoiesis: expression of SDF-1 by mesothelial cells and biliary ductal plate epithelial cells. Proc Natl Acad Sci U S A (1999) 96(15):8585–90. 10.1073/pnas.96.15.8585 10411919 PMC17560

[6] Feng Y Broder CC Kennedy PE Berger EA HIV-1 entry cofactor: functional cDNA cloning of a seven-transmembrane, G protein-coupled receptor. Science (1996) 272(5263):872–7. 10.1126/science.272.5263.872 8629022

[7] Oberlin E Amara A Bachelerie F Bessia C Virelizier JL Arenzana-Seisdedos F The CXC chemokine SDF-1 is the ligand for LESTR/fusin and prevents infection by T-cell-line-adapted HIV-1. Nature (1996) 382(6594):833–5. 10.1038/382833a0 8752281

[8] Bleul CC Farzan M Choe H Parolin C Clark-Lewis I Sodroski J The lymphocyte chemoattractant SDF-1 is a ligand for LESTR/fusin and blocks HIV-1 entry. Nature (1996) 382(6594):829–33. 10.1038/382829a0 8752280

[9] Crump MP Gong JH Loetscher P Rajarathnam K Amara A Arenzana-Seisdedos F Solution structure and basis for functional activity of stromal cell-derived factor-1; dissociation of CXCR4 activation from binding and inhibition of HIV-1. EMBO J (1997) 16(23):6996–7007. 10.1093/emboj/16.23.6996 9384579 PMC1170303

[10] Arenzana-Seisdedos F Virelizier JL Rousset D Clark-Lewis I Loetscher P Moser B HIV blocked by chemokine antagonist. Nature (1996) 383(6599):400 10.1038/383400a0 8837769

[11] Amara A Gall SL Schwartz O Salamero J Montes M Loetscher P HIV coreceptor downregulation as antiviral principle: SDF-1alpha-dependent internalization of the chemokine receptor CXCR4 contributes to inhibition of HIV replication. J Exp Med (1997) 186(1):139–46. 10.1084/jem.186.1.139 9207008 PMC2198965

[12] Colin P Benureau Y Staropoli I Wang Y Gonzalez N Alcami J HIV-1 exploits CCR5 conformational heterogeneity to escape inhibition by chemokines. Proc Natl Acad Sci USA (2013) 110(23):9475–80. 10.1073/pnas.1222205110 23696662 PMC3677469

[13] Alkhatib G Locati M Kennedy PE Murphy PM Berger EA. HIV-1 coreceptor activity of CCR5 and its inhibition by chemokines: independence from G protein signaling and importance of coreceptor downmodulation. Virology (1997) 234(2):340–8. 10.1006/viro.1997.8673 9268166

[14] Jin J Colin P Staropoli I Lima-Fernandes E Ferret C Demir A Targeting spare CC chemokine receptor 5 (CCR5) as a principle to inhibit HIV-1 entry. J Biol Chem (2014) 289(27):19042–52. 10.1074/jbc.M114.559831 24855645 PMC4081942

[15] Agace WW Amara A Roberts AI Pablos JL Thelen S Uguccioni M Constitutive expression of stromal derived factor-1 by mucosal epithelia and its role in HIV transmission and propagation. Curr Biol (2000) 10(6):325–8. 10.1016/S0960-9822(00)00380-8 10744978

